# Hippocampal resting‐state connectivity is associated with posterior‐cortical cognitive impairment in Parkinson's disease

**DOI:** 10.1002/brb3.3454

**Published:** 2024-03-12

**Authors:** Dana Pourzinal, Jihyun Yang, Katie L. McMahon, David A. Copland, Leander Mitchell, John D. O'Sullivan, Gerard J. Byrne, Nadeeka N. Dissanayaka

**Affiliations:** ^1^ Faculty of Medicine The University of Queensland Centre for Clinical Research Herston Australia; ^2^ School of Clinical Sciences, Faculty of Health Queensland University of Technology Brisbane Australia; ^3^ Queensland Aphasia Research Centre, School of Health and Rehabilitation Sciences The University of Queensland Brisbane Australia; ^4^ Surgical Treatment and Rehabilitation Service (STARS) Education and Research Alliance The University of Queensland and Metro North Health Queensland Australia; ^5^ School of Psychology The University of Queensland St Lucia Australia; ^6^ Department of Neurology Royal Brisbane & Women's Hospital Herston Australia; ^7^ Mental Health Service Royal Brisbane & Women's Hospital Herston Australia

**Keywords:** cognitive impairment, hippocampus, Parkinson's disease, resting‐state FMRI

## Abstract

**Aim:**

Frontal and posterior‐cortical cognitive subtypes in Parkinson's disease (PD) present with executive/attention and memory/visuospatial deficits, respectively. As the posterior‐cortical subtype is predicted to progress rapidly toward dementia, the present study aimed to explore biological markers of this group using resting‐state functional magnetic resonance imaging (rs‐fMRI).

**Methods:**

*K*‐means cluster analysis delineated subtypes (cognitively intact, frontal, posterior‐cortical, and globally impaired) among 85 people with PD. A subset of PD participants (*N *= 42) and 20 healthy controls (HCs) underwent rs‐fMRI. Connectivity of bilateral hippocampi with regions of interest was compared between posterior‐cortical, cognitively intact, and HC participants using seed‐based analysis, controlling for age. Exploratory correlations were performed between areas of interest from the group analysis and a series of cognitive tests.

**Results:**

The posterior‐cortical subtype (*N *= 19) showed weaker connectivity between the left hippocampus and right anterior temporal fusiform cortex compared to the cognitively intact (*N *= 11) group, *p‐*false discovery rate (FDR) = .01, and weaker connectivity between bilateral hippocampi and most fusiform regions compared to HCs (*N *= 20). No differences were found between HCs and cognitively intact PD. Exploratory analyses revealed strongest associations between connectivity of the right anterior temporal fusiform cortex and left hippocampus with category fluency (*p‐*FDR = .01).

**Conclusion:**

Results suggest that weakened connectivity between the hippocampus and fusiform region is a unique characteristic of posterior‐cortical cognitive deficits in PD. Further exploration of hippocampal and fusiform functional integrity as a marker of cognitive decline in PD is warranted.

## Graphical abstract

Hippocampal resting‐state connectivity in Parkinson's disease cognitive subtypes

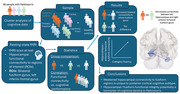

## 1 INTRODUCTION

Although motor symptoms are the major clinical hallmark of Parkinson's disease (PD), cognitive impairment is a prevalent feature (Aarsland et al., 2021). The heterogeneity of cognitive symptoms observed in PD has led to the development of cognitive subtypes. Subtyping promotes early identification of those at risk of dementia, allowing for the development of biomarkers and targeted preventative treatments (Greenland et al., 2019). The dual syndrome hypothesis proposes that there are two distinct cognitive syndromes in PD; a posterior‐cortical syndrome presenting with memory and visuospatial deficits rooted in posterior (temporal, parietal, and occipital) dysfunction and a frontal syndrome presenting with executive and attentional deficits stemming from fronto‐striatal dysfunction (Kehagia et al., 2013). It is the posterior‐cortical syndrome that is predicted to decline more rapidly than the frontal syndrome in terms of global cognitive ability (Williams‐Gray et al., 2013). However, research into the biological and clinical markers of those with posterior‐cortical impairments is limited.

Given that memory impairment is indicative of posterior‐cortical deficits, a potential biological marker for posterior‐cortical dysfunction may be the compromised integrity of the hippocampus. Associations between both functional and structural integrity of the hippocampus and episodic memory function in PD have been reported (Pourzinal et al., 2021). In particular, resting‐state functional magnetic resonance imaging (rs‐fMRI) studies have found reduced connectivity between the hippocampus and regions such as the inferior frontal gyrus (Hou et al., 2016), occipital gyrus (Yao et al., 2016), and precuneus (Bezdicek et al., 2019) to be related to poorer performance on verbal and visual episodic memory tasks.

The present study therefore aimed to compare resting state functional connectivity of the hippocampus between posterior‐cortical impaired PD and control participants. Given the nature of posterior‐cortical deficits, hippocampal connectivity with select regions of the brain related to episodic memory was explored as regions of interest (ROIs). It was predicted that functional connectivity between the hippocampus and regions related to episodic memory would be compromised in the posterior‐cortical subtype compared to cognitively intact people with PD and healthy controls (HCs). An exploratory post hoc analysis was also performed correlating significant connectivities from the group analysis with various cognitive tests to determine their sensitivity to the specific ROIs.

## 2 METHODS

### 2.1 Participants

The present study used existing data available from our previous work, which identified four cognitive subtypes in a sample of 85 PD participants using the machine learning technique called *k*‐means clustering (Pourzinal et al., 2020). All participants were recruited from movement disorders clinics and/or community settings (e.g., support groups and community organizations) in Brisbane, Australia from 2016 to 2018. PD participants met the UK Brain Bank criteria for PD (Gibb & Lees, 1988). Participants were excluded if they scored <20 on the Montreal Cognitive Assessment, which is the cut‐off point for PD dementia (Biundo et al., 2014), had self‐reported major depression or anxiety disorder, or were ineligible for MRI (e.g., pacemaker and claustrophobia). HCs meeting criteria for mild cognitive impairment (MCI) were also excluded (see Section 2.3). Only a subset of eligible PD participants (*N* = 42) and HCs (*N *= 21) who underwent rs‐fMRI were included in the present study. All participants were scanned while on their usual PD medications, and written informed consent was received from each participant. Ethical approval was obtained from the University of Queensland and the Royal Brisbane and Women's Hospital HREC committees prior to commencement.

### 2.2 Measures

Self‐report measures were collected via a questionnaire and included basic demographic and clinical information, as well as the Geriatric Depression Scale (GDS) (Sheikh & Yesavage, 1986) and Geriatric Anxiety Inventory (GAI) (Matheson et al., 2012) to measure depression and anxiety, respectively. Participants then participated in a face‐to‐face interview where the Montreal Cognitive Assessment was administered to measure global cognition (Nasreddine et al., 2005) and the Movement Disorders Society Unified Parkinson's disease Rating Scale (MDS‐UPDRS) was administered as a measure of parkinsonism. The total MDS‐UPDRS score reflected overall parkinsonism and part three (MDS‐UPDRS‐III) reflected motor symptom severity only (Goetz et al., 2007). Levodopa equivalent daily dose was calculated using the method cited in Tomlinson et al. (2010).

### 2.3 Neurocognitive assessment

All participants also underwent comprehensive neurocognitive assessment adhering to level 2 of the *Movement Disorder Society* guidelines for identification of PD–MCI (Litvan et al., 2012). The following tests were administered: two tests within the memory (Hopkins Verbal Learning Test‐Revised delayed recall; Brief Visuospatial Memory Test‐Revised delayed recall) (Benedict & Brandt, 2007) language (Delis‐Kaplan Executive Function System (D‐KEFS) category fluency total score; (Delis et al., 2004) Boston Naming Test (Kaplan et al., 1983)), executive function (D‐KEFS card‐sorting sort recognition score; (Delis et al., 2004) Trail Making Test B (Lezak, 2012)), visuospatial (CLOX; (Royall et al., 1998) Benton's Judgment of Line Orientation (Calamia et al., 2011)), and attention/working memory (Trail Making Test A; (Lezak, 2012) STROOP (Golden & Freshwater, 2002) color‐word scale) domains were administered. Adjusted *z*‐scores were derived using age and/or education based normative scores, and participants who scored less than 1.5 *SD* on two or more tests met criteria for MCI.

### 2.4 Cluster analysis

The clustering methodology used to delineate cognitive subtypes and sample characteristics for each subtype have been described elsewhere (Pourzinal et al., 2020). Briefly, *k*‐means cluster analysis was performed on ten cognitive measures categorized into “frontal” and “posterior‐cortical” cluster variables based on previous literature. Four groups of participants with unique patterns of cognitive impairment across the frontal and posterior measures were identified: (1) globally impaired; (2) posterior‐cortical impaired; (3) frontal impaired; and (4) cognitively intact. To investigate potential biomarkers for rapid cognitive decline, the present study focuses on functional connectivity of the posterior‐cortical impaired subtype compared to the cognitively intact subtype and HCs. However, all subtypes were included in the exploratory analyses.

### 2.5 Imaging acquisition and preprocessing

Participants were scanned at the Herston Imaging Research Facility with a Siemens 3T Prisma scanner and 20‐channel head coil. Standard MRI safety checks were performed by both study investigators and imaging facility staff. High‐resolution 3‐dimensional T1‐weighted MP2RAGE scans were acquired (TE = 2.05 ms, TI = 700 ms, FA = 3, and field of view = 256 × 240 × 176 mm^3^). During the 8‐min resting‐state session, participants were instructed to remain still, close their eyes, and think of nothing as 260 BOLD sensitive gradient EPI were acquired (TE = 30 ms, TR = 2050 ms, flip angle = 80°, axial slices = 50, slice thickness = 2.6 mm, interslice gap = 0 mm, and field of view = 190 × 190 mm^2^; Greenland et al., 2019). All preprocessing steps, including realignment, co‐registration, normalization (MNI space), smoothing (GK = 6 mm; Kehagia et al., 2013), scrubbing (global bold signal change >5 *SD* or motion parameter displacement >0.9 mm), and denoising, were performed using CONN toolbox 20b (http://www.nitrc.org/projects/conn), SPM 12 (Wellcome Trust Centre for Neuroimaging, London, UK), and MATLAB 2020b. Anatomical component‐based noise correction modeling was performed to mitigate confounding effects of white matter, cerebrospinal areas, and motion. Temporal frequencies outside of 0.008–0.09 Hz were removed to minimize physiological, head‐motion, and other noise sources (Weissenbacher et al., 2009). Head motion >2.6 mm (1 voxel) in any direction resulted in exclusion of the participant.

### 2.6 Imaging analysis

Imaging analysis was performed using the CONN toolbox. An ROI–ROI analysis was performed to explore differences in functional connectivity of the hippocampus between groups. All ROIs were defined using the default Harvard–Oxford atlas in CONN. Bilateral hippocampi, left inferior frontal gyri (opercularis and triangularis), and bilateral fusiform cortices (anterior, posterior, and occipital) were selected as ROIs based on a meta‐analysis that identified these brain regions, among others, to be most consistently related to episodic memory recall in healthy young adults using a subsequent memory approach (Kim, 2011). First‐level analyses were performed by applying a generalized linear model to identify significant temporal correlations between ROIs while regressing out motion.

### 2.7 Statistical analysis

Clinical and cognitive measures were compared between groups using parametric (*t*‐test, ANOVA) and nonparametric (Wilcoxon, Mann–Whitney *U*, and Phi and Cramer's *V*) tests depending on violation of parametric assumptions across variables, at a significance threshold of Bonferroni‐*p* < .05. All statistical comparisons were performed using SPSS 27.0.1.0. Group‐level rs‐fMRI analyses were performed controlling for age to compare hippocampal functional connectivity of the posterior‐cortical impaired group to the cognitively intact and HC groups. A connection significance threshold of *p* < .01 (false discovery rate (FDR) corrected) and ROI significance threshold of *p *< .05 (FDR corrected; MVPA omnibus test) was used. Hippocampal connectivity with areas of interest from the group analysis were then correlated with adjusted cognitive *z*‐scores from the full PD–MCI test battery using spearman's correlation in R version 3.6.3 and R studio version 1.4.1717. All subtypes were included in the correlation analysis, including the globally impaired (*N *= 8) and frontal impaired (*N *= 3) subtypes, and analyses were performed both with and without HCs. FDR‐adjusted *p* < .05 was used to correct for multiple comparisons; however, due to the exploratory nature of the post hoc analyses, uncorrected *p*‐values were also reported. Significant results from the exploratory analysis were then adjusted for age using partial correlations.

## 3 RESULTS

### 3.1 Data checks

Two participants, from the posterior‐cortical impaired and HC groups, were excluded from the imaging analysis due to excessive head motion (>2.6 mm). A mean of 2.58 (*SD* = 3.58, range = 0–14) volumes were censored per participant. There were no missing data within the neurocognitive dataset. Scores were missing from MDS‐UPDRS, education, and GAI scales. However, due to the low quantity of missing data overall (<10% per measure), missing data were handled by available case analysis (Bennett, 2001).

### 3.2 Participants

Sample characteristics for the total PD (all subtypes with rs‐fMRI data) and HC samples are provided in Table 1, with PD participants demonstrating slightly higher depression (GDS) scores than controls. Sample characteristics for the group functional connectivity analysis (cognitively intact, posterior‐cortical impaired, and HC groups) are provided in Table 2. In terms of demographic variables, the posterior‐cortical impaired group was older than cognitively intact and HC groups and exhibited slightly higher depression scores than HCs. In terms of cognitive variables, the posterior‐cortical impaired group performed significantly worse than both control groups on visual delayed episodic memory and category fluency and performed significantly worse than HCs on verbal episodic memory and attentional/executive measures (STROOP and Trail‐Making A and B). Cognitively intact PD and HCs did not differ in terms of demographics or cognition.

**TABLE 1:** Sample characteristics for the total Parkinson's disease (PD) and healthy control (HC) samples (mean ± SD).

Variable	PD (*N*=41)		HC (*N*=20)		Test statistic	*p*‐FDR
	Mean/*N*	*SD*	Mean/*N*	*SD*		
Age (year)	68.78	8.66	63.90	7.84	*T = .254*	–
Sex (*female*)	17 (41%)		10 (50%)		*Φ = −.081*	–
Education (year)	12.95	3.59	14.69	3.18	*T = .296*	–
MoCA	24.98	2.76	26.45	2.16	*T = .166*	–
GAI	1.82	4.14	.45	.99	*U = 447*	–
GDS	2.59	1.94	.95	.15	U = 623*	.001
Disease duration (year)	4.73	3.45	–	–	–	–
MCI	13 (30%)		–	–	–	–
LEDD (mg)	485.33	290.94	–	–	–	–
MDS‐UPDRS Total	35.22	13.83	–	–	–	–
MDS‐UPDRS Motor	17.59	10.00	–	–	–	–
Hoehn and Yahr			–	–	–	–
*1*	23 (56%)		–	–	–	–
*2*	15 (37%)		–	–	–	–
*3*	3 (7%)		–	–	–	–

Abbreviations: GAI, Geriatric Anxiety Inventory; GDS, geriatric depression scale; LEDD, levodopa equivalent daily dose; MDS‐UPDRS, Movement Disorder Society Unified Parkinson's Disease Rating Scale; MoCA, Montreal Cognitive Assessment; MCI, mild cognitive impairment.

*p < .05.

**TABLE 2:** Comparison of sample and cognitive characteristics for group analysis (mean ± SD).

Demographic variable	Group						Test statistic	*p*‐Bonferroni
	PC: Posterior‐cortical (*N *= 19)		CI: Cognitively intact (*N *= 11)			HC: Healthy controls (*N *= 20)		
	Mean/*N*	*SD*	Mean/*N*	*SD*	Mean/*N*	*SD*		
Age (year)	71.30	8.76	60.10	5.24	63.90	7.84	F = 8.16*	PC vs. HC = .013 PC vs. CI = .002
Sex (female)	11 (57%)		8 (75%)		10 (50%)		*Φ* = .150	–
Education (year)	12.68	3.37	15.10	2.13	14.69	3.18	*F = 2.83*	–
MoCA	24.70	2.60	27.00	2.05	26.45	2.16	F = 4.32*	PC vs. CI = .042
GAI	2.50	5.44	1.44	2.35	.45	.99	*H = 2.008*	–
GDS	2.45	1.73	2.50	2.37	.95	.15	H = 9.043*	PC vs. HC = .012
Dis. duration (year)	4.60	3.02	5.00	3.33	–	‐	*U* = 112.0	–
MCI	7 (37%)		1 (9%)		–	–	*Φ* = −.267	–
LEDD (mg)	458.75	266.62	631.13	384.09	–	–	*U* = 122.0	–
MDS–UPDRS Total	34.11	15.79	29.33	9.87	–	–	*U* = 66.0	–
MDS–UPDRS Motor	17.30	9.86	10.50	6.40	–	–	*U* = 57.0	–
Hoehn and Yahr					–	–	*Φ* = .316	–
*1*	12 (63%)		9 (81%)		–	–	–	–
*2*	6 (32%)		1 (9%)		–	–	–	–
*3*	1 (5%)		0		–	–	–	–
**Cognitive variable**	*z‐*Score	*SD*	*z‐*Score	*SD*	*z‐*Score	*SD*	**Test statistic**	***p*‐Bonferroni**
**Memory**								
Visual: BVMT	−.74	1.18	.76	.58	.80	1.17	F = 8.92*	PC vs. HC = < .001 PC vs. CI = .016
Verbal: HVLT	.15	.77	1.06	.40	.32	.91	F = 5.21*	PC vs. HC = < .048
**Language**								
Category fluency	−.22	.90	1.11	1.29	.97	1.30	F = 7.12*	PC vs. HC = .005 PC vs. CI = .012
Boston naming	−.36	.77	.00	.68	.04	.89	*F = 1.56*	–
**Executive**								
Card sorting	−.28	.93	1.09	.83	1.08	.86	*F = 3.46*	–
TMT‐B	.32	.67	.79	.98	.97	.69	F = 3.27*	PC vs. HC = .044
**Visuospatial**								
CLOX	.40	.99	.91	.55	.48	1.07	*H = 2.14*	–
JLO	.11	.87	.03	.93	.27	.86	*H = .529*	–
**Attention/WM**								
TMT‐A	.32	.67	.79	.98	.93	.68	F = 4.31*	PC vs. HC = .017
STROOP	−.46	.70	−0.11	.56	.64	1.38	H = 8.83*	PC vs. HC = .009

Abbreviations: BVMT, Brief Visuospatial Memory Test (delayed); Dis., disease; GAI, Geriatric Anxiety Inventory; GDS, Geriatric Depression Scale; HVLT, Hopkin's Verbal Learning Test (delayed); JLO, Judgment of Line Orientation; LEDD, levodopa equivalent daily dose; MoCA, Montreal Cognitive Assessment; MCI, mild cognitive impairment; MDS‐UPDRS, Movement Disorder Society Unified Parkinson's Disease Rating Scale; TMT, Trail‐Making Test; WM, working memory.

*p < .05 *Note*.

### 3.3 Between‐groups ROI–ROI functional connectivity analysis

Significant results for each ROI are detailed in Table 3. There was a significant difference in functional connectivity between PD groups such that the left anterior temporal fusiform cortex showed significantly weaker connectivity to the right hippocampus in the posterior‐cortical impaired group compared to the cognitively intact group, *p‐*FDR = .009. There were no statistically significant differences in hippocampal functional connectivity between the cognitively intact group and HCs. The posterior‐cortical impaired group demonstrated consistently weaker bilateral hippocampal connectivity with all subdivisions of the fusiform gyrus compared to HCs, except between the left hippocampus and left anterior temporal fusiform cortex.

**TABLE 3:** Between‐groups functional connectivity analysis results.

	Group comparison					
	Cognitively intact (*N *= 11) vs. posterior‐cortical (*N *= 19)		Cognitively intact (*N *= 11) vs. healthy controls (*N *= 20)		Healthy controls (*N *= 20) vs. posterior‐cortical (*N *= 19)	
ROI	Hip (L)	Hip (R)	Hip (L)	Hip (R)	Hip (L)	Hip (R)
Anterior temporal FC (L)	*ns*	*ns*	*ns*	*ns*	*ns*	*T *= 3.56, *p* = .002
Anterior temporal FC (R)	*T *= −3.28, *p* = .009	*ns*	*ns*	*ns*	*T* = 4.47, *p* < .001	*T *= 3.61, *p* = .002
Posterior temporal FC (L)	*Ns*	*ns*	*ns*	*ns*	*T *= 4.90, *p* < .001	*T *= 4.46, *p* < .001
Posterior temporal FC (R)	*ns*	*ns*	*ns*	*ns*	*T* = 4.52, *p* < .001	*T* = 5.31, *p* < .001
Temporo‐occipital FC (L)	*ns*	*ns*	*ns*	*ns*	*T *= 5.30, *p* < .001	*T *= 4.74, *p* < .001
Temporo‐occipital FC (R)	*ns*	*ns*	*ns*	*ns*	*T *= 5.59, *p* < .001	*T *= 4.14, *p* < .001
Occipital FC (L)	*ns*	*ns*	*ns*	*ns*	*T *= 6.06, *p* < .001	*T *= 4.38, *p* < .001
Occipital FC (R)	*ns*	*ns*	*ns*	*ns*	*T* = 5.45, *p* < .001	*T *= 3.51, *p* = .002
IFG opercularis (L)	*ns*	*ns*	*ns*	*ns*	*ns*	*ns*
IFG triangularis (L)	*Ns*	*ns*	*ns*	*ns*	*Ns*	*ns*

Abbreviations: FC, fusiform cortex; Hip, hippocampus; IFG, inferior frontal gyrus; ns, nonsignificant; *p*, FDR‐adjusted *p*‐value; ROIs, regions of interest.

### 3.4 Exploratory correlation analysis

The strength of functional connectivity between the bilateral hippocampus with the right anterior temporal fusiform cortex showed significant correlations with cognitive *z*‐scores from the PD–MCI battery. The results conveyed in Table 4a show that, within the PD sample, right anterior temporal fusiform cortex connectivity to the left hippocampus correlated with delayed verbal episodic memory, category fluency, and card sorting, whereas connectivity with the right hippocampus correlated only with delayed verbal episodic memory. However, none of these results remained significant after adjustment for multiple comparisons. When HCs were added to the sample (Table 4b), the positive correlation between category fluency and right anterior temporal fusiform connectivity to the left and right hippocampus was strengthened, remaining significant after multiple comparison adjustment. However, when controlling for age, the partial correlation between category fluency and right anterior temporal fusiform connectivity to the left (*r* = .23, *p* = .06) and right (*r* = .15, *p* = .24) hippocampus was no longer significant, with the left hippocampus just above the significance threshold.

**TABLE 4:** Correlation between hippocampal functional connectivity values and cognitive tests for Parkinson's disease (PD) sample and whole sample.

4a. PD only (*N *= 41)							
		Hip (L)—atFC (R)				Hip (R)—atFC (R)	
		*r*‐Value	Unadj‐*p*	Adj‐*p*	*r*‐Value	Unadj‐*p*	Adj‐*p*
Memory	Visual: BVMT	.26	.10	.30	.02	.88	.94
	Verbal: HVLT	.34	.03*	.19	.38	.01*	.11
Language	Category fluency	.33	.03*	.20	.28	.07	.27
	Boston naming	.09	.58	.75	.10	.54	.71
Executive function	Card sorting	.31	.05*	.22	.29	.06	.24
	TMT‐B	.17	.29	.54	.24	.13	.34
Visuospatial	CLOX	.13	.31	.59	−.01	.12	.97
	JLO	−.02	.22	.94	−.14	.68	.59
Attention/WM	TMT‐A	.16	.40	.54	.25	.96	.31
	STROOP	.19	.90	.46	.07	.36	.85
**4b. Whole sample (*N *= 61)**							
Memory	Visual: BVMT	.21	.10	.38	.10	.43	.30
	Verbal: HVLT	.17	.20	.79	.25	.05	.51
Language	Category fluency	.39	.00*	.01*	.36	.00*	.03*
	Boston naming	.07	0.60	.75	.11	.39	.60
Executive function	Card sorting	.23	.07	.22	.14	.29	.48
	TMT‐B	.12	.38	.59	.14	.28	.47
Visuospatial	CLOX	.14	.27	.45	.10	.44	.62
	JLO	.02	.87	.91	−.07	.58	.75
Attention/WM	TMT‐A	.06	.65	.79	.11	.39	.60
	STROOP	.20	.13	.31	.19	.15	.34

Abbreviations: atFC, anterior temporal fusiform cortex; BVMT, brief visual memory test (delayed); HVLT, Hopkins Verbal Learning Test (delayed); Hip, hippocampus; JLO, Judgment of Line Orientation; TMT, Trail‐Making Test; WM, working memory.

*p < 05 (unadj, unadjusted *p* value; adj, FDR‐adjusted *p*‐value).

## 4 DISCUSSION

### 4.1 Summary of results

The present study aimed to explore functional connectivity of the hippocampus in people with PD with posterior‐cortical deficits in a novel attempt to discover potential rs‐fMRI markers of increased dementia risk in PD. We found that the posterior‐cortical subtype had weaker connectivity at rest between the right anterior temporal fusiform cortex and left hippocampus than the cognitively intact subtype. Hippocampal connectivity to almost all fusiform regions was weaker in posterior‐cortical PD participants compared to HCs, whereas there were no significant differences between HCs and cognitively intact PD participants. These results support the hypothesis that hippocampal connectivity to memory‐related areas would be compromised in those with posterior‐cortical impairments compared to controls. Exploratory correlation analyses also revealed that category fluency positively correlated with connectivity between the right anterior temporal fusiform cortex and bilateral hippocampus, with only the left hippocampus remaining marginally significant after controlling for age.

### 4.2 Current findings in the context of Alzheimer's literature

To date, no studies have explored rs‐fMRI markers of the posterior‐cortical subtype in PD. However, the results of the present study align with findings reported in the rs‐fMRI literature in MCI related to Alzheimer's disease (AD–MCI). Decreased functional connectivity between the right fusiform gyrus and left parahippocampal gyrus at rest has been detected in people with AD–MCI with memory impairments compared to HCs (Cai et al., 2015). The strength of this connectivity also positively correlated with global cognitive ability (mini–mental state exam). Two additional studies found that people with AD–MCI with memory impairment exhibited reduced connectivity between the right fusiform gyrus and right hippocampus compared to HCs; however, correlations with cognitive measures were not revealed (Chen et al., 2016; Wang et al., 2011). In context of the literature, results from the present study, therefore, suggest that compromised hippocampal connectivity to the right anterior temporal fusiform gyrus may be a marker of memory impairment.

### 4.3 Contralateral compensatory mechanisms in PD

The exploratory analysis also revealed a positive association between left hippocampal connectivity with the right anterior temporal fusiform cortex and category fluency, a measure of language and semantic memory. Although this result was only marginally significant after controlling for age, this was likely due to the limited statistical power of the present study and may reflect a true association beyond the effects of age. This calls into question the lateralization of language function, which is conventionally thought to be dominated by the left hemisphere (Ocklenburg & Güntürkün, 2018). Functional MRI studies have demonstrated that greater task‐dependent activity in the left hippocampus and left fusiform gyrus (Gaillard et al., 2003), as well as stronger functional connectivity between the two regions at rest (Pihlajamäki et al., 2000), correlated with higher category fluency scores in general adult populations. So why would functional connectivity of the right anterior temporal fusiform cortex relate to category fluency function in PD?

One possibility may be that, akin to AD (Thompson et al., 2003), atrophy of the left hemisphere may precede the right, resulting in a compensatory mechanism achieved by recruiting contralateral regions. However, it is unclear whether atrophy occurs in a systematic left to right fashion in PD. Using measures of cortical thickness, Claassen et al. (2016) revealed greater left hemisphere atrophy in early‐stage PD and greater right hemisphere atrophy in late‐stage PD. Another study revealed that, although people with PD–MCI had lower baseline left hippocampus volume compared to HCs and cognitively intact PD, they exhibited greater atrophy of the right hippocampus over four years (Mak et al., 2015). Hanganu et al. (2014) also found that, although people with PD–MCI showed extensive whole‐brain atrophy over a two‐year period, cognitively intact people with PD had increased cortical thinning in only the left fusiform and left lateral occipital regions compared to controls. Greater gray matter loss in the left fusiform gyrus than the right in PD dementia compared to PD without dementia has also been reported, suggesting more profound left‐hemisphere atrophy (Burton et al., 2004). Taken together, these studies defend the left‐to‐right pattern of atrophy in PD.

Whether this atrophy relates to function is not clear. From the AD literature, Pineault et al. (2018) found that whereas task‐related activity in the left fusiform gyrus during category fluency was similar for memory‐impaired people with AD–MCI and HCs, task‐related activity in the right fusiform gyrus and its positive correlation with task performance was unique to the memory‐impaired AD–MCI group (Pineault et al., 2018). In the PD literature, Mak et al. (2015) found increased right fusiform gyrus activity during an fMRI facial perception task compared to HCs, potentially compensating for left hemispheric dysfunction (Cardoso et al., 2010). Our previous work also revealed increased right angular gyrus activity in people with PD–MCI compared to HCs and cognitively intact people with PD during an alternating category fluency task, indicating additional recruitment of the contralateral hemisphere in PD with cognitive impairment (Yang et al., 2022). While increased activity in the right hemisphere during category fluency has been reported with respect to normal ageing (Meinzer et al., 2009), the aforementioned studies highlight PD‐specific compensatory activity over and above that of age‐matched controls. Ultimately, further multi‐model MRI studies combining structural and functional techniques are needed to better understand compensatory mechanisms in PD.

### 4.4 Implications

Reduced functional connectivity between fusiform areas and hippocampi in posterior‐cortical impaired people with PD compared to control groups suggests that these regions play a pivotal role in posterior‐cortical subtype. This aligns well with the predictions of the dual syndrome hypothesis, which suggests that posterior‐cortical cognitive symptoms may stem from compromised cholinergic pathways involving subcortical and temporal regions of the brain, among others (Kehagia et al., 2013). Hippocampal connectivity with the right anterior temporal fusiform cortex appears uniquely impacted in posterior‐cortical impaired participants compared to controls. Further exploration of this compromised connectivity as a biomarker for the posterior‐cortical subtype is warranted, given the pragmatic value of developing a rs‐fMRI marker of future cognitive decline in PD. The clinical utility of rs‐fMRI, which can be administered in a short 15‐min session while the patient is at rest, is much greater compared to task‐based fMRI, which often requires lengthier sessions with active participation from the patient. It is also advantageous over structural MRI, which cannot provide information about functionality of the brain. Additionally, the results speak to the potential compensatory mechanisms that arise in PD–MCI. In terms of lateralized function, the results may support the concept of contralateral compensation for category fluency tasks before severe cognitive decline. Again, further research on this topic is critical in order to determine the relevance of compensatory functional connectivity at rest to cognitive ability in specific domains.

Finally, the findings highlight the critical role of the fusiform cortex in posterior‐cortical impairments in PD. The role of the fusiform gyrus in both verbal episodic and semantic memory is well‐established (Palacio & Cardenas, 2019) and was reaffirmed in the present study for people with PD. However, this region is also known for its role in visual processing (Palejwala et al., 2020). It is therefore plausible that the memory and visuospatial deficits of the posterior‐cortical syndrome may arise from altered fusiform functionality. Although the posterior‐cortical subtype demonstrated visual memory impairment in the present study, no correlations were revealed between visual memory or other visuospatial measures and fusiform‐hippocampal connectivity values. However, studies have linked verbal memory, semantic memory, visuospatial impairments to brain atrophy, and cognitive decline in PD (Pelletier et al., 2017; Pourzinal et al., 2021). Thus, although the role of the hippocampus in episodic memory and cognitive decline in PD is well‐established (Pourzinal et al., 2021), further exploration of the functional and structural integrity of the fusiform gyrus as a potential biomarker for cognitive decline in PD is necessary.

### 4.5 Strengths and limitations

A major strength of the present study was the use of data‐driven subtyping to separate the posterior‐cortical subtype from the cognitively intact. Badea et al. (2017) found that rs‐fMRI results were neither replicated across nor within PD datasets due to vast clinical heterogeneity. In this sense, homogenizing the data using cognitive subtypes was favorable. Employing cognitively intact PD participants as controls was also advantageous, as this controlled for certain PD‐related confounds, such as PD pathology, levodopa intake, motor symptomatology, and disease duration. Comparisons between the posterior‐cortical and frontal impaired subtypes could not be made due to small sample size, warranting future studies to establish specificity of hippocampal rs‐fMRI connectivity as a biomarker. Overall sample size was also limited, restricting the number of covariates applied and potentially masking results due to low power. However, in compensation for the small sample, ROI–ROI analyses were performed as opposed to whole‐brain analysis, reducing the number of statistical tests performed and thus type 1 error.

Another limitation was the absence of important covariates in the analyses, such as sex, education, dopaminergic/non‐dopaminergic medications, which were omitted due to limited sample size and risk of overfitting (Soch et al., 2016). Levodopa intake is particularly important to account for given its influence on rs‐fMRI signals in PD (Yang et al., 2016) and may thus have confounded the results. Age may also provide an alternative explanation for the results, given that increased age is related to greater cognitive impairment in PD (Aarsland et al., 2021), and the posterior‐cortical group was older than the control groups. However, all analyses were adjusted for age to minimize this effect. Finally, the results may lack generalizability due to the unique data‐driven subtyping methodology.

## 5 CONCLUSIONS

Connectivity between the right hippocampus and left anterior temporal fusiform cortex was compromised in posterior‐cortical impaired people with PD compared to cognitively intact PD and HC participants, suggesting that dysfunction of this connectivity may be pivotal to the behavioral profile of the posterior‐cortical subtype. Weaker strength of this connectivity also correlated with poorer category fluency, which is potentially suggestive of a compensatory mechanism in action. Although exploratory in nature, the results of the correlation analysis justify further research into hippocampal connectivity with the fusiform region as a biomarker for the identification and targeted treatment of cognitive decline in PD.

## AUTHOR CONTRIBUTIONS

**Dana Pourzinal**: Conceptualization; methodology; writing—original draft; writing—review and editing; formal analysis; visualization; investigation. **Jihyun Yang**: Methodology; software; validation; supervision; writing—review and editing; project administration; conceptualization. **Katie L. McMahon**: Methodology; supervision; writing—review and editing; project administration. **David A. Copland**: Methodology; project administration; writing—review and editing; supervision. **Leander Mitchell**: Methodology; writing—review and editing; resources. **John D. O'Sullivan**: Methodology; writing—review and editing; supervision. **Gerard J. Byrne**: Methodology; funding acquisition; writing—review and editing; supervision. **Nadeeka N. Dissanayaka**: Methodology; writing—review and editing; supervision; resources; project administration; funding acquisition; conceptualization.

## CONFLICT OF INTEREST STATEMENT

The authors report there are no conflicts of interest to declare.

## IRB AND CONSENT STATEMENT

All participants provided informed, written consent prior to participation in the study, and the protocol received approval from the Royal Brisbane and Women's Hospital (HREC/10/QRBW/446) and University of Queensland (UQ2015001881) Human Research Ethics Committees.

### PEER REVIEW

The peer review history for this article is available at https://publons.com/publon/10.1002/brb3.3454.

## Data Availability

Data may be available upon request.
